# Choline and Working Memory Training Improve Cognitive Deficits Caused by Prenatal Exposure to Ethanol

**DOI:** 10.3390/nu9101080

**Published:** 2017-09-29

**Authors:** Jaylyn Waddell, Sandra M. Mooney

**Affiliations:** Department of Pediatrics, University of Maryland School of Medicine, 655 W. Baltimore Street, BRB 13-059, Baltimore, MD 21201, USA; Sandra.mooney@som.umaryland.edu

**Keywords:** delayed non-matching to place, fetal alcohol spectrum disorder, nutrition, cognitive flexibility, T-maze

## Abstract

Prenatal ethanol exposure is associated with deficits in executive function such as working memory, reversal learning and attentional set shifting in humans and animals. These behaviors are dependent on normal structure and function in cholinergic brain regions. Supplementation with choline can improve many behaviors in rodent models of fetal alcohol spectrum disorders and also improves working memory function in normal rats. We tested the hypothesis that supplementation with choline in the postnatal period will improve working memory during adolescence in normal and ethanol-exposed animals, and that working memory engagement during adolescence will transfer to other cognitive domains and have lasting effects on executive function in adulthood. Male and female offspring of rats fed an ethanol-containing liquid diet (ET; 3% *v*/*v*) or control dams given a non-ethanol liquid diet (CT) were injected with choline (Cho; 100 mg/kg) or saline (Sal) once per day from postnatal day (P) 16–P30. Animals were trained/tested on a working memory test in adolescence and then underwent attentional set shifting and reversal learning in young adulthood. In adolescence, ET rats required more training to reach criterion than CT-Sal. Choline improved working memory performance for both CT and ET animals. In young adulthood, ET animals also performed poorly on the set shifting and reversal tasks. Deficits were more robust in ET male rats than female ET rats, but Cho improved performance in both sexes. ET male rats given a combination of Cho and working memory training in adolescence required significantly fewer trials to achieve criterion than any other ET group, suggesting that early interventions can cause a persistent improvement.

## 1. Introduction

Fetal alcohol spectrum disorder (FASD) is an umbrella term used to describe the range of disabilities caused by prenatal alcohol exposure. Recent data estimates that 2–5% of children may have a FASD [1,2]. Children and adults with FASD perform poorly in many tasks that assess executive function [3,4,5,6,7,8,9]. The term executive function describes efficient interaction between many cognitive domains, such as allocation of attention, impulse control or response inhibition, mental flexibility and working memory [10,11]. Successful engagement of these domains supports the ability to keep information ‘in mind’, attend to appropriate cues and update information as contingencies change. Though many studies have documented deficits in executive function evident in FASD, few studies have attempted to improve these deficits with cognitive domain-specific training despite positive outcomes [12,13].

Choline supplementation during the perinatal period improves a number of cognitive outcomes in typically developing rats, and these improvements endure well into adulthood. Robust working and reference memory improvements, evidenced by fewer errors and increased memory capacity, were apparent in adult rats given perinatal choline supplementation [14,15]. Early choline supplementation also improves complex problem-solving tasks in which ambiguous cues predict escape only in particular configurations with other cues [16]. Prenatal choline supplementation also reduced impulsivity and enhanced accuracy of interval timing in adulthood [17]. Choline supplementation can also improve cognitive outcomes in children with FASD. Nutritional supplements during pregnancy appear to protect against some of the teratogenic effects of in utero alcohol exposure in infants (e.g., [18]). Nutrition is typically poor in women who drink heavily during pregnancy. A multivitamin and mineral supplement, including B vitamins, folic acid, calcium, iron and zinc with or without the addition of choline was administered to pregnant women reporting alcohol consumption during pregnancy, or the supplement was merely recommended in a non-supplemented control group [18]. Nutritional supplementation that included choline was effective in improving memory, visual stimulus processing and response latency in infants [18]. Effects of the multivitamin and mineral supplement alone were not reliable. This study underscores the specific benefit of choline in the prevention of behavioral deficits in infants exposed to alcohol in utero.

The essential nutrient choline was first demonstrated to ameliorate learning and cognition deficits in a rodent model of FASD [19]. When given during gestation, choline reduces developmental delays, such as delayed eye opening, and improves behavior in rat pups exposed to ethanol [20]. The role of choline in brain development is multifaceted, as it is necessary for membrane formation, methyl group donation and as a precursor of acetylcholine [21,22]. Dietary choline availability influences DNA methylation (e.g., [23]) and choline given pre- and postnatally can enhance cholinergic activity in the brain throughout the lifespan [14,24,25,26,27,28]. The beneficial window of choline supplementation is quite prolonged, ranging from prenatal to early adolescence in rats [14]. Choline is well tolerated in children and improved a cognitive task involving the hippocampus as well as global cognitive function scores in children with FASD supplemented between 2.5–5 years of age [29,30]; but see [31].

Working memory is a component of executive function that choline can improve in normal and ethanol exposed rats [14,19,25,32,33]. Working memory is the ability to retain and manipulate information in short periods of time [34]. Efficient and accurate updating requires working memory with sufficient capacity and duration. It is a strong predictor of cognitive abilities, such as problem solving and reasoning as well as academic outcomes (e.g., [35,36]). Normal working memory ability relies on the frontal cortices [37,38] and spatial working memory requires cooperation between the prefrontal cortex and hippocampus [38,39,40,41]. Rats exposed to prenatal ethanol exhibit deficits in spatial working memory in adolescence, even with very short delays, and deficits at longer delays persist into adulthood [42]. In humans, working memory matures during adolescence, as accuracy and processing speed dramatically increase between 12–15 years of age [43,44]. Working memory deficits are evident in adolescents with FASD [45]. During adolescence in rodents, the prefrontal cortices undergo significant development and structural refinement (e.g., [46,47]). In humans, working memory tasks activate subregions of the frontal cortices, and better working memory performance is associated with greater activation of neural networks involved in more general executive function [40].

The study presented here tested the hypothesis that working memory training during adolescence combined with choline supplementation would improve cognitive flexibility in a rat model of FASD and do so more effectively than either intervention alone. To do this, male and female rat pups exposed to ethanol during gestation or not, were treated with choline between P16–P30. Choline supplementation at this time can produce long-lasting improvement in spatial working memory, reducing the number of errors made in a radial arm maze when tested much later in adulthood [14]. During the adolescent period, rats were trained on a difficult but attainable delayed non-matching to place task that increased the working memory delay as rats became capable of stable response accuracy. Rats were then tested in an attentional set shifting and reversal learning task to assess cognitive flexibility and executive function in young adulthood (e.g., [48]). We tested cognitive flexibility in adulthood to determine whether the benefits of choline, working memory training or both might endure and generalize to a more complex task. Though choline alone improved working memory training and some aspects of cognitive flexibility in young adulthood, the combination of choline and working memory training was most effective in male rats. Our results suggest that combining nutritional support and cognitive domain-specific training can be more efficacious than either alone.

## 2. Methods

Timed pregnant Long-Evans rats were purchased from Envigo (Frederick, MD, USA) and arrived on gestational day (G)3. Animals were housed at the University of Maryland School of Medicine in an Association for Assessment and Accreditation of Laboratory Animal Care International (AAALAC) accredited facility. The facility is kept on a 12/12 h light/dark cycle (lights on at 7 a.m.) and is controlled for humidity (40–45%) and temperature (22 °C). All procedures performed were in accordance with the guidelines for animal care established by the National Institutes of Health as well as with the approval of the Institutional Animal Care and Use Committee (IACUC) at the University of Maryland School of Medicine (protocol 1214005).

Nine dams were assigned to the control (CT) group and 10 dams were assigned to the ethanol-exposed (ET) group. All dams were given ad libitum access to a nutritionally complete liquid diet (by American Institute of Nutrition (AIN-93) standards; L10251A, Research Diets, New Brunswick, NJ, USA) from G6 until G20 similar to prior reports from our laboratory (e.g., [49,50,51]) with the exception that ET dams were given a diet containing 3% ethanol for the duration of the exposure. We consider this to be a mild model of FASD. This concentration is similar to that used by others (3.35%) which was determined to produce a relatively constant blood alcohol concentration of 31 mg/deciliter [52]. CT animals received an alcohol-free isocaloric, isonutritive liquid diet containing maltose-dextrin in place of the ethanol calories. All animals received fresh food daily, no feeding tubes were ever completely emptied, and the volume of diet consumed was recorded each day (see Figure S1). Litters were culled to 10–12 pups within 24 h of birth with equal distribution of males and females when possible. Pups were not cross-fostered at parturition as is common in rodent models of prenatal ethanol exposure. Maternal care can be poor in dams administered an alcohol diet during gestation, but we do not observe any maternal deficits. Other laboratories have also found that maternal behavior is not compromised following ethanol exposure during gestation, but this methodological difference is noted, as it could influence behavioral outcomes [53].

Pups were treated with daily subcutaneous injections of saline (0.9% Sal; pH 7.4) or choline (Cho; 100 mg/kg; Sigma-Aldrich, St. Louis, MO, USA) once per day from postnatal day (P)16 until P30. Care was taken to reduce stress during injections, but it is noted that this treatment regimen likely induces at least temporary stress. Pups were assigned to either a group receiving working memory training (Tr) or an untrained group (Utr) that was exposed to the T-maze (described below) without training from P30 through P38. Thus, offspring were assigned to one of four postnatal groups: Sal-Utr; Sal-Tr; Cho-Utr; Cho-Tr. Only one male and one female from any litter was assigned to a group. The number of rats in each condition is listed in Table 1.

Prior to the attentional set shifting and reversal learning task, females were staged for estrus by vaginal swab daily beginning on P55. Acclimation to digging for reward and simple discrimination was conducted in estrus between P60–P65, so that the CD, IDSs, and EDS were conducted in diestrus. This was done to avoid testing during proestrus. We anticipated that the reversal phase and extradimensional shift might be frustrating or stressful. Females are particularly vulnerable to negative effects on learning when stress occurs during proestrus (e.g., [54]). Because this generated an age range at which animals are used, males were yoked; thus, if a female began the task at P62, a male in the same experimental condition also began at that age.

Delayed Non-Matching to Place: Animals were food restricted beginning on P28 and shaped to approach food reward bowls in a T-maze on P29. The T-maze was built using the dimensions published and effective for testing rats [55]. Rats were trained on the delayed non-matching to place task (DNMP) between P30 and P38. Each training trial consisted of two phases. The first is referred to as a demonstration trial in which one arm of the maze is blocked, leaving one arm open. After the rat consumed the reward in the open arm, it was placed in the start position again for a pre-determined delay. After the delay, the barrier was removed and the rat was permitted to choose between both the left and right arm. The rat was reinforced when it chose the arm not previously visited in the immediately preceding demonstration trial. The rat must maintain the previous response ‘in mind’ and choose the other arm to obtain a reward. The task was run over eight days with 12 trials per day; the first five daily sessions used a 10 s delay. This was the point at which the majority of rats reached a criterion of 75% correct in a session. The task then progresses, and the next two sessions used a 30 s delay to increase the difficulty of the task. The final day was done with 0 s delay to ensure that animals had learned the non-match rule. It should be noted that training with a 0 s delay is typically conducted at the beginning of training. The insertion of the delay after the rule “go the other way” is established more clearly tests working memory duration or capacity than the way we conducted our experiment. Our primary intent was to challenge rats during this developmental phase, and to keep the task challenging through the juvenile phase of development, but this methodological difference should be noted.

Untrained animals were exposed to the T-maze for the same number of trials as those undergoing DNMP training but both bowls contained a reward. Thus, these rats experienced the T-maze but no demands were placed on working memory. This group was included to rule out the possibility that daily handling and exposure to the maze could constitute environmental enrichment and improve behavior generally, rather than an effect of working memory engagement during adolescence. All animals were returned to ad libitum access to chow on P38. The number of correct trials and percent correct was recorded for each trained animal. Latency to reach the reward bowl was recorded for all animals.

Attentional Set Shifting and Reversal Learning: The attentional set shifting test method was based on the adaptation for use in rodents [48]. Animals were tested in the series of discriminations and reversal phase between P58 and P70 in a plexiglass chamber with an open top (49 cm length, 39 cm wide, 24 cm high) under dim light. In the attentional set shifting task rats progressed through a series of discriminations in which they dug in one of two small bowls for food reward. For all phases, the number of trials to criterion (TTC; i.e., the number of trials each rat required to achieve 8 correct choices in a row) was recorded. This task used six phases: Phase 1 consisted of a simple discrimination (SD), in which only the odor cue differed between the bowls. One odor (e.g., garlic) predicted the presence of reward, and another (cloves) predicted the absence of reward. On phase 2, another dimension was added; odor continued to predict which bowl was baited with reward but a second dimension of digging media (e.g., stones or feathers) was varied, and must be learned to be irrelevant (compound discrimination; CD). Rats then progressed to an intradimensional shift (IDS) where odor still predicted reward, however, the specific odors and non-predictive dimensions changed; now odor 3 (cinnamon) predicted presence and odor 4 (rosemary) predicted absence of reward. Once the criterion was achieved, the contingencies were reversed (IDS reversal, IDS-R). Animals then performed a second IDS (IDS2), still with odor as the predictive dimension to ensure that normal rats formed an attentional set to the dimension of odor (e.g., [56]). An attentional set is formed when an organism attends to an aspect of a multidimensional stimulus that has been associated with or predictive of reward. To determine whether rats formed a set for odor, an extradimensional shift (EDS), in which the predictive stimulus changed from odor to the digging media (e.g., sponge vs. beads), was conducted. If rats form an attentional set, they require more trials to achieve the criterion in this phase, as they require more error feedback to make this predictive shift. In normal rats, both reversal learning and performance on the EDS phase will require more trials for criterion than the phase immediately prior to each. Thus, shift cost [shift cost = TTC(EDS) − TTC(IDS2)] will be positive in normal rats.

## 3. Statistical Analysis

The mean and standard error of the mean were calculated. Mean diet consumption, total number of pups and percent male were compared for CT and ET animals using independent samples *t*-tests. Statistical analysis was conducted using SigmaPlot (Version 12, Systat, San Jose, CA, USA) and SPSS (Version 18, IBM, Armonk, NY, USA). For all behavioral outcomes, males and females were analyzed separately to retain sufficient power to detect within-sex group effects. The percent number of trials correct for each session of DNMP training was calculated for each rat and the mean and standard error was computed for each group. These values were analyzed by repeated measures analysis of variance (ANOVA) with session as the within subjects variable and prenatal treatment (CT or ET) and postnatal treatment (Sal or Cho) as between group factors and session as the within group factor. Data from rats in the untrained condition were not included in analysis, as this group did not have a working memory demand, and therefore their responses could not be considered correct or incorrect.

Analysis of the attentional set shifting and reversal learning task was conducted on the average number of trials required to achieve 8 consecutive correct responses in each phase and the average number of errors made in each phase. Repeated measures ANOVA were conducted with testing phase (6) as the within subjects factor and prenatal (CT or ET) and postnatal treatment (Sal or Cho) and DNMP training (Tr or Utr) as between groups factors. Each prenatal group was further analyzed independently with repeated measures ANOVA to determine whether group differences were present based on the postnatal manipulations. Specifically, the effect of phase x group was tested to determine when choline or choline with working memory training might produce differences that were not detected in the larger ANOVA, due to the large number of groups. This ANOVA was followed by Bonferroni post hoc tests. In cases where groups were found to differ, independent samples *t*-tests were used to determine which phases were different between these groups. The EDS shift cost was calculated and analyzed by ANOVA with prenatal, postnatal and training condition as between subjects factors.

## 4. Results

Dam and Litter Outcomes: A total of 9 dams were used in the CT groups, providing 62 offspring. Ten dams produced 64 pups for the ET groups. Pregnant rats consumed on average 96.97 ± 1.42 mL of diet per day with no difference (*t*(17) = −0.0931; *p* > 0.05) between the CT and ET groups (96.84 ± 2.02 and 97.1 ± 2.08 mL per day, respectively; see Supplemental Data). The average ethanol intake was 2.38 ± 0.38 g/day. Litters had a mean of 11.16 ± 0.47 pups with no significant difference (*t*(17) = 0.343, *p* > 0.05) between CT (11.33 ± 0.58 pups per litter) and ET (11 ± 0.76 pups per litter). Similarly, the percent of males was not significantly different between groups (CT litters contained 41 ± 7.1% males and ET litters had 52 ± 4.8% males; *t*(17) = −1.317; *p* > 0.05). Pup weights did not significantly differ between the CT and ET rats for either sex, nor were there significant differences in weight between saline and choline treated pups, or any significant interactions, largest *F* = 1.06.

### 4.1. Delayed Non-Matching to Place

**Figure 1:**
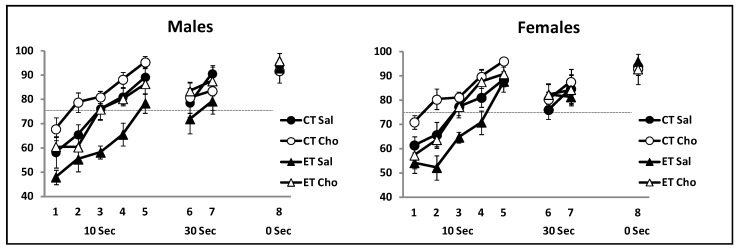
Delayed non-matching to place acquisition data for male and female rats trained on P30–P38. Rats were trained in a T-maze with one 12 trial session each day. The first five daily sessions used a 10 s delay between the demonstration and test trial. The majority of rats reached a criterion of 75%, indicate 3d by the dashed line, by day 5. After this the delay was increased to 30 s for two sessions. Rats were then tested with no retention delay to confirm acquisition of the non-matching rule. Each data point represents the Mean ± SEM. CT = Control, ET = Ethanol; Sal = Saline, Cho = Choline. *n* = 7–9 per group.

Repeated measures ANOVA assessing the 8 sessions of T-maze training confirmed a significant effect of session, as all groups increased the number of correct responses across days, *F*(7, 182) = 30.81, *p* < 0.0001; Figure 1, left panel). Both the main effect of prenatal treatment (CT or ET), *F*(1, 26) = 12.81, *p* < 0.001 and postnatal treatment (Saline or Choline), *F*(1, 26) = 11.46, *p* < 0.002 were significant. No significant interactions were detected, largest *F* = 1.12. As depicted in Figure 1, ET rats required more training to attain at least 75% correct in a session.

Repeated measures ANOVA, with the percent trials correct for each session as the dependent variable and prenatal and postnatal treatments as independent variables, found a significant effect of session, *F*(7, 196) = 33.99, *p* < 0.001, as all groups increased the number of correct responses across sessions (Figure 1, right panel). A significant prenatal × session interaction was detected, *F*(7, 196) = 2.48, *p* < 0.01. This interaction is likely due to the fact that choline facilitated acquisition in ET rats, and this group surpassed saline treated CT rats. The main effect of prenatal treatment (CT or ET) was significant, *F*(1, 28) = 9.26, *p* < 0. 005, as well as the main effect of postnatal treatment (Saline or Choline), *F*(1, 28) = 12.95, *p* < 0.001. The prenatal × postnatal interaction was not significant, *F* < 1.

### 4.2. Attentional Set Shifting and Reversal Learning

**Figure 2:**
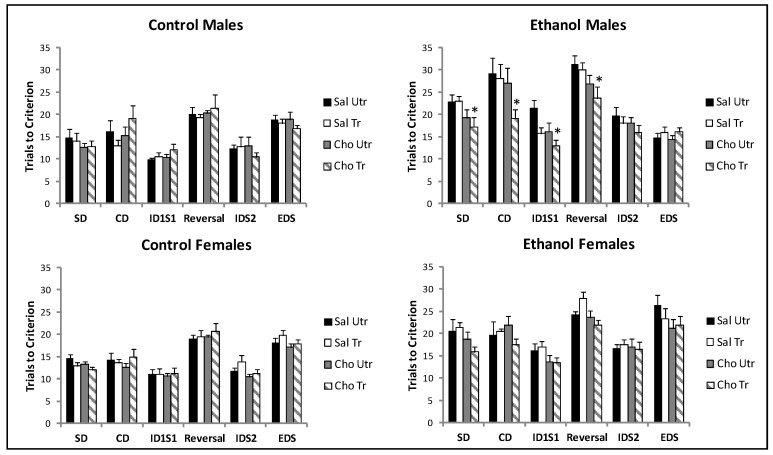
Attentional set shifting and reversal learning in young adult rats. In most phases, male and female rats prenatally exposed to ethanol required more trials to reach criterion than control rats. Choline supplementation reduced the number of trials in some phases in both sexes. The combination of working memory training and choline was more effective than either alone in males. Bars represent the Mean ± SEM, *n* = 7–9 per group. * Denotes a significant difference compared to Sal Utr rats in the same prenatal condition. CT = Control, ET = Ethanol; Sal = Saline, Cho = Choline; Utr = No Working Memory training, Tr = Working Memory training.

Trials to Criterion: Analysis of data generated by males using repeated measures ANOVA was conducted on the total number of trials required for each rat to achieve 8 correct responses in a row for each phase (6) with prenatal treatment (CT or ET), postnatal treatment (Sal or Cho) and DNMP training (Utr or Tr) as between subjects variables. The results are presented in the left panel of Figure 2. A significant interaction between the testing phase and prenatal treatment was found, *F*(5, 270) = 13.93, *p* < 0.0001. Ethanol exposure changed the pattern of training trials necessary to achieve the criterion compared to CT rats. No other interactions reached significance. A significant between subjects effect of prenatal treatment *F*(1, 54) = 63.53, *p* < 0.0001 as well as postnatal treatment was detected, *F*(1, 54) = 5.64, *p* < 0.021. An interaction between prenatal treatment and postnatal treatment was also significant, *F*(1, 54) = 7.65, *p* < 0.008. Male ET rats required more trials to achieve criterion than CT rats in most phases, and choline reduced trials to achieve the criterion in ET rats. To further determine whether choline or choline with working memory training improved performance within each prenatal condition, repeated measures ANOVA was conducted on CT males and ET males separately. This analysis confirmed that neither intervention improved performance in CT males. The main effect of phase was significant, *F*(5, 130) = 24.36, *p* < 0.0001, but neither the phase × group interaction or main effect of group was significant, *F*s < 1. The effect of phase was significant in ET males as well, *F*(1, 140) = 32.98, *p* < 0.0001, but the phase x group interaction was not significant, *F* = 1.25, *p* > 0.05. The main effect of group was significant, *F*(3, 28) = 4.87, *p* < 0.0008. Bonferroni post hoc comparisons confirmed that the only group significantly different from the Sal-Utr group in the ET prenatal condition was the Cho-Tr group, *p* < 0.006. Independent samples *t*-tests were conducted to compare the Sal-Utr group to the Cho-Tr group for each phase to determine which testing phases were improved by the combined interventions. Ethanol exposed male Cho-Tr rats were significantly different than Sal-Utr rats in the SD, *t*(14) = 2.17, *p* < 0.04, CD, *t*(14) = 2.4, *p* < 0.03, IDS1, *t*(14) = 3.85, *p* < 0.002, and the reversal phase, *t*(14) = 2.24, *p* < 0.02. This pattern of results suggests that the combination of choline supplementation and DNMP training was most effective in facilitating discrimination performance and reversal learning, at least in males.

Analysis of the number of trials to achieve criterion required for females to complete each phase was conducted with repeated measures ANOVA for each phase (6) with prenatal treatment (CT or ET), postnatal treatment (Sal or Cho) and DNMP training (Tr or Unt) as between subjects variables. No significant interactions were detected. However, a significant between subjects effect of prenatal treatment *F*(1, 56) = 99.51, *p* < 0.0001, and postnatal treatment was detected, *F*(1, 56) = 8.44, *p* < 0.005 (Figure 2, right panel). Female ET rats required more trials to reach criterion in each phase and choline improved performance. Repeated measures ANOVA conducted on each prenatal condition separately confirmed that neither choline nor choline combined with working memory training changed performance in control females. The effect of phase was significant, *F*(5, 135) = 54.73, *p* < 0.0001, but neither the phase x group or main effect of group was significant, largest *F* = 1.03. Similarly, in ethanol exposed females, the effect of phase was significant, *F*(5, 140) = 19.69, *p* < 0.0001 but the phase x group interaction was not significant, *F* < 1. The between subjects factor of group was significant, *F*(1, 28) = 3.81, *p* < 0.02. The Cho-Tr group was significantly different compared to the Sal-Tr group, *p* < 0.02. These groups differed in both the SD, *t*(15) = 3.49, *p* < 0.003 and reversal phase (15) = 3.30, *p* < 0.005. This is likely driven by the high number of trials to criterion evident in the Sal-Tr group.

**Figure 3:**
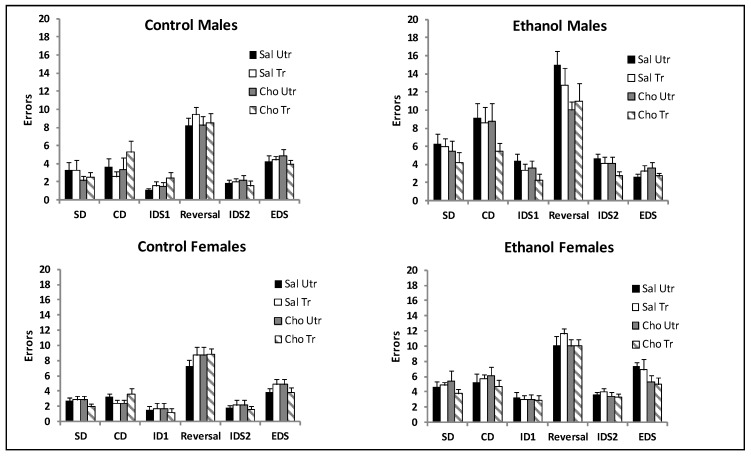
Number of errors made in each phase of the attentional set shifting and reversal task. Male and female rats exposed to ethanol made more errors in most phases than control rats. Ethanol exposed male rats made significantly more errors in the reversal phase, and choline significantly reduced errors. Bars represent the Mean ± SEM, *n* = 7–9 per group. Abbreviations as in Figure 2.

Errors: Repeated measures ANOVA was conducted on the total number of errors for each phase (6) with prenatal treatment (CT or ET), postnatal treatment (Sal or Cho) and DNMP training (Tr or Utr) as between subjects variables in males and females separately (Figure 3). The analysis confirmed a testing phase x prenatal condition interaction, *F*(5, 270) = 8.72, *p* < 0.0001 in males. No other interactions with the testing phase were significant, largest *F*(5, 270) = 1.44, *p* > 0.05. The main effect prenatal condition was significant, *F*(1, 54) = 38.4, *p* < 0.0001 as well as the prenatal x postnatal interaction, *F*(1, 54) = 4.24, *p* < 0.04. No other interactions were significant, largest *F*(1, 54) = 2.99, *p* > 0.05. The number of errors made during the reversal phase were analyzed by ANOVA with prenatal treatment (CT or ET), postnatal treatment (Sal or Cho) and DNMP training (Utr or Tr) as between subjects variables. This phase typically captures perseveration, as the rat continues to respond to the specific stimulus that predicted reward in the previous phase. The effect of prenatal treatment was significant, *F*(1, 54) = 13.73, *p* < 0.001, indicating that male ET rats made more perseverative errors than male CT rats. The effect of choline was also significant, *F*(1, 54) = 3.97, *p* < 0.05, indicating that choline reduced perseverative errors. This appears to be driven by the reduction evident in the male ET groups (Figure 3).

Analysis of data from female rats failed to detect significant interactions between testing phase and any between subject variables (Figure 3, right panel). The main effect of prenatal treatment was significant, *F*(1, 56) = 70.38, *p* < 0.0001, indicating that ET females made significantly more errors across testing phases than CT females. No interactions were significant, largest *F*(1, 56) = 3.07, *p* < 0.08. The number of errors made during the reversal phase was analyzed by ANOVA with prenatal treatment (CT or ET), postnatal treatment (Sal or Cho) and DNMP training (Utr or Tr) as between subjects variables. The main effect of prenatal treatment was significant, *F*(1, 56) = 12.82, *p* < 0.001. No other factors or interactions were significant.

**Figure 4:**
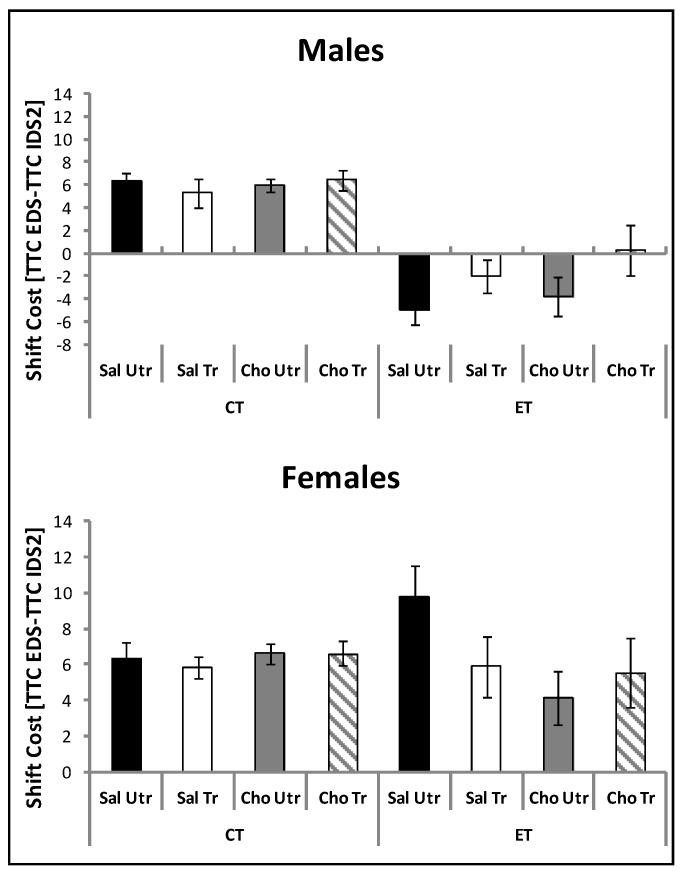
EDS shift cost for each group. ET males (top panel) did not form an attentional set, and this was not reversed by choline, working memory training or the combination of the two. Females (bottom panel) formed an attentional set irrespective of prenatal or postnatal conditions. Bars represent the Mean ± SEM, *n* = 7–9 per group. Abbreviations as in Figure 2.

Shift Cost: The EDS shift cost was calculated (TTC EDS-TTC IDS2) and analyzed using ANOVA with prenatal condition (CT or ET), postnatal condition (Sal or Cho) and DNMP training (Tr or Utr) as between group factors for male and female rats separately. In male rats, the main effect of prenatal condition was significant, *F*(1, 54) = 75.62, *p* < 0.001 (Figure 4). No other main effect reached significance, largest *F*(1, 54) = 3.61, *p* > 0.05. Male CT rats required more trials to shift their attention from odor to media compared to their performance in the IDS2 immediately preceding the EDS. This indicates that male CT rats formed an attentional set during the previous phases of testing. This was not true for male ET rats. The majority of ET rats did not exhibit an increase in the number of trials necessary to reach criterion between IDS2 and the EDS regardless of postnatal interventions.

In females, no significant group differences were detected, largest *F*(1, 56) = 3.33, *p* < 0.07 (Figure 4). In contrast to males, female ET rats did exhibit the typical increase in trials when the predictive dimension changed from odor to media, and this increase was similar to that of CT female rats. This increase is considered normal, as it demonstrates that females had acquired knowledge of the perceptual set of odor. The fact that males exhibited no such increase suggests they used a simpler strategy in each phase that did not rely on formation of a set.

## 5. Discussion

Prenatal ethanol exposure disrupted performance in a spatial working memory and cognitive flexibility task in male and female rats. Choline robustly improved performance in both CT and ET rats trained on a spatial working memory task in adolescence, replicating effects of prenatal ethanol exposure reported by others in this task (e.g., [42]). Though ET rats were slower to reach response criterion in the DNMP task, they were capable of performing the task at both the short and longer working memory delay. This aligns with findings from other laboratories using an escalating dose of alcohol that reaches higher concentrations than used in the current study [57,58]. Choline-treated ET rats performed similarly to saline treated CT rats in both sexes. This result confirms that choline supplementation from P16–P30 can improve working memory in both normal and ethanol exposed rats, and that the effective developmental window of choline administration might be quite protracted in ET rats as it is in normal rats [14,59]. The prefrontal cortices are critical for successful performance in the DNMP task, and prenatal ethanol disrupts normal immediate early gene activation of these regions by engagement in a working memory task [57]. Fetal alcohol exposure also reverses the effects of cholinergic drugs on measures of working memory compared to rats never exposed to alcohol [58]. Our results suggest that choline can ameliorate these effects and improve performance in a task that requires cooperation between the frontal cortices and other brain regions (discussed below).

Ethanol effects on cognitive flexibility (i.e., attentional set shifting and reversal learning) were more robust in male rats. ET females required significantly more trials to complete some phases of the task compared to same sex CT rats, but their reversal and EDS performance was similar to that of same sex CT rats. Males, however, made more errors during the reversal phase and failed to make an attentional set. Choline was effective in reducing the number of trials necessary to reach criterion in both sexes as well, though the deficits were sexually dimorphic. The results suggest that choline supplementation may have promoted generalization of working memory improvements to a test of cognitive flexibility in male rats. Prenatal ethanol exposure disrupts social interaction and engagement of the frontal cortices in male rats during adulthood more so than females [60,61]. Interestingly, choline improved working and reference memory more in male rats than female rats in a radial arm maze task [62]. Our observation of more global impairments in males permits more obvious benefit from the interventions used here.

Choline supplementation can metabolically imprint changes in brain regions necessary for working memory and higher order cognitive function. Muscarinic receptor density is increased in both the hippocampus and frontal cortex in adult rats [14,63]. A high choline diet during pregnancy increased nicotinic receptor density in these same brain regions of the offspring [64]. Prenatal choline can decrease levels of acetylcholinesterase, increasing availability of acetylcholine in the developing hippocampus [65]. Both the morphology and distribution of neurons within the frontal cortices, hippocampus and septum have also been observed in adults following perinatal choline supplementation [62]. The availability of choline directly influences cell membrane and synapse formation, contributing to the synthesis of the dominant phospholipid phosphatidylcholine [66]. The effects of choline on brain development are also through epigenetic mechanisms, as choline is a significant methyl donor (reviewed in [22]). It is through its epigenetic effects that choline alters the epigenome of neuronal and endothelial progenitors (reviewed in [22]). Thus, the effects of choline in the developing brain are diverse, contributing to its various behavioral benefits through a long developmental window.

Cholinergic systems in the brain have a diffuse neuromodulatory role and are involved in multiple behaviors contributing to adaptive executive function, including attention, plasticity, and learning and memory [67,68,69]. During cortical development acetylcholine acts as a trophic factor, shaping neural networks and synchronizing communication between the prefrontal cortex and the hippocampus [70]. Cholinergic projections from the basal forebrain innervate the prefrontal cortex and hippocampus in early postnatal development in rodents [71,72]. These projections are critically engaged during working memory tasks, particularly tasks with a spatial component [73,74]. Within the striatum, tonically active cholinergic interneurons are largely responsible for acetylcholine release and cholinergic tone [75]. The prefrontal cortex and striatum are part of a cortico-striato-thalamo-cortical loop necessary for cognitive flexibility, and this loop is modulated by cholinergic activity [76].

Acetylcholine release within the dorsomedial striatum is critical for efficient response adaptation in place reversal learning [77]. Acetylcholine levels increase in the dorsomedial striatum during strategy switches and inhibition of this increase impairs place reversal learning [77]. Antagonism of M1 muscarinic receptors in this region increases errors on response reversal learning but not initial learning, highlighting the necessity of cholinergic modulation when learned contingencies change [78]. Animal models of FASD show impaired reversal learning in a number of motivational systems, including appetitive [79,80] and defensive (i.e., avoidance or escape) learning [81,82]. Choline improved learning in a spatial discrimination reversal task in rats prenatally exposed to ethanol [32]. Male rats exposed to prenatal ethanol made significantly more errors during the reversal phase in a non-spatial discrimination here, and this was reversed by choline. It is possible that choline supplementation enhances or restores cholinergic activity during reversal learning in ethanol exposed rats.

An attentional set is formed when an organism attends to an aspect of a multidimensional stimulus that has been associated with reward. When the predictive dimension changes, more experience with the stimulus dimensions is necessary to ignore the previously acquired rule. In primates, the lateral prefrontal cortex [83,84] and in rodents, the medial prefrontal cortex [48,85] is required to efficiently shift away from this set when the dimension controlling the contingency changes. In adult lesion studies, ablation of the prefrontal cortex dramatically increases the number of trials required to reach criterion relative to the most recent intradimensional shift, indicating that the prefrontal cortex is critical for updating information when contingencies change [48,83,85]. The functional conservation of the frontal cortices from primates to rodents is also evident in the role of the orbital frontal cortex in reversal learning. Lesions of the orbitofrontal cortex dramatically increase perseverative errors in reversal of discrimination learning [83,86,87]. Interestingly, orbital frontal cortex lesions can also abolish formation of an attentional set [87,88]. Rather than cause an increase in the number of trials to make the extradimensional shift, the number remains about the same as the preceding IDS [87,88]. Lesions of the dorsomedial striatum also increase errors in the reversal phase and abolish formation of an attentional set [89]. The pattern observed here in ethanol exposed males parallels that reported after orbitofrontal or dorsomedial striatum lesions. It is possible that prenatal ethanol exposure disrupts communication between the frontal cortices and the striatum.

The neural substrates recruited in children with FASD differ from those of non-exposed children in tasks that require aspects of executive function such as response inhibition [90]. Children with FASD exhibited more general activation of the frontal cortices than non-exposed children [90]. In typically developing children, the frontal cortices exhibit very selective and task specific patterns of activation [44,90]. Working memory and attention training in children with FASD produced positive results, improving working memory and reducing distractibility [12]. Generalization to academic performance was evident, as the training improved reading fluency [12]. Self-regulation training also generalized to other domains of executive function in children with FASD, improving inhibitory control and social cognition [13]. Thus, accumulating evidence suggests that children with FASD can improve in many domains with either experiential or nutritional interventions. The results presented here suggest that cognitive domain specific training may generalize more effectively when coupled with nutritional support.

Further experiments are necessary to determine the stability of generalization and longevity of beneficial effects of combined nutritional intervention and the specificity of working memory training. Our experiment did not train rats on the rule governing reinforcement in the DNMP task prior to insertion of a working memory delay. This was done to make the task more difficult, but might have changed the demands in early phases as the rats had to discern the rule, and maintain their previous response across a delay. Using more difficult working memory tasks might improve the robustness of the effects seen here. It is possible that male rats exposed to ethanol would form an attentional set if more discriminations were used in the task. Indeed, the number of intradimensional shifts can influence the strategy used to solve the phases of the attentional set shifting task (e.g., [88]). It is also possible that adding more perceptual dimensions, or changing the sensory modality of the dimensions could change the pattern of results in either sex. This is an important limitation of the current study. Tasks that model cognitive impairments in both sexes are necessary. Here, we found that performance in ethanol exposed males deviated more from control males than ethanol exposed females deviated from control females. The fact that males are more impaired is not supported by clinical data in FASD populations [91]. Though brain morphological findings favor females [92], male and female children exhibit cognitive and social deficits [91,92]. More complex cognitive tasks may be necessary to detect deficits in females, or higher doses of ethanol may be necessary to induce more robust deficits in females in the task used here.

## 6. Conclusions

Mild prenatal ethanol exposure resulted in long term cognitive deficits in rats that were typically more apparent in males than females. Intervention during adolescence in the form of nutritional supplementation with choline and training on a working memory task was effective in males, and the combination was more effective than either intervention alone. Thus, our results suggest that the benefits of experience and nutritional therapy might be synergistic, supporting generalization of cognitive domain training. Further work is necessary to determine whether the interventions are efficacious at different developmental phases, and how well working memory training generalizes to other cognitive domains. These results further support a beneficial effect of choline in FASD, and suggest it may more efficacious when coupled with cognitive training.

## Figures and Tables

**Table 1:** Number of animals per group.

Experimental Group	Male Control	Male Ethanol	Female Control	Female Ethanol
**Saline Untrained**	8	8	8	8
**Saline Trained**	7	8	8	8
**Choline Untrained**	8	8	8	7
**Choline Trained**	7	8	8	9

## Supplementary Materials

The following are available online at www.mdpi.com/2072-6643/9/1080/s1, Figure S1: Average liquid diet consumption for Ad Lib control dams and ethanol exposed dams. There were no significant differences in amount consumed.
