# Supplementary material for: Choline and Working Memory Training Improve Cognitive Deficits Caused by Prenatal Exposure to Ethanol
Source: Nutrients. 2017 Sep 29;9(10):1080. doi: 10.3390/nu9101080 (PMC5691697; doi:10.3390/nu9101080)
Supplement: Supplementary file 1 [file nutrients-09-01080-s001.docx]

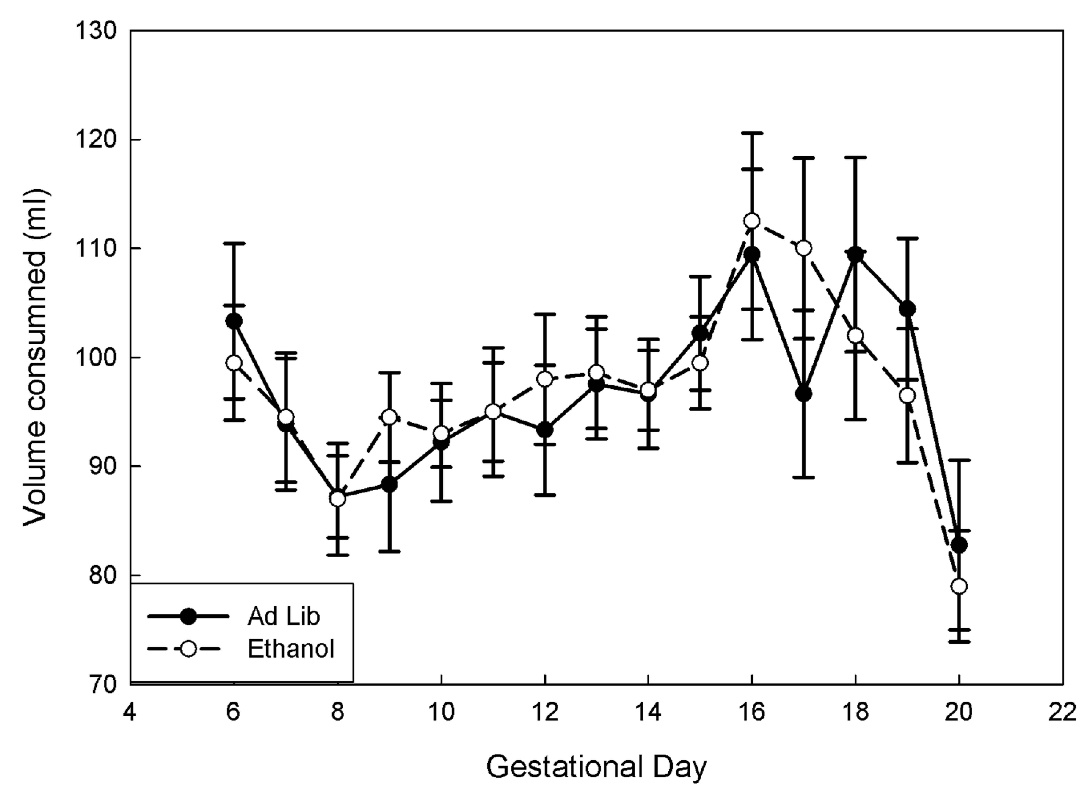


Figure S1. Average liquid diet consumption for Ad Lib control dams and ethanol exposed dams. There were no significant differences in amount consumed.
